# Skin Basal Cell Carcinosarcoma in a Trichoblastic Guise—Molecular Characterization of a Novel Entity Mimicking a Pyoderma Gangrenosum

**DOI:** 10.3390/dermatopathology13030045

**Published:** 2026-09-16

**Authors:** Paul Georg, Roland Blum, Robert Hunger, Bastian Dislich, Ronald Wolf

**Affiliations:** 1 Department of Dermatology, Inselspital, University Hospital Bern, Anna-Seiler-Allee 33, 3010 Bern, Switzerland; paul.georg@hin.ch (P.G.); roland.blum@medicspathologie.ch (R.B.); re.hunger@gmx.net (R.H.); 2 Institute of Tissue Medicine and Pathology, University of Bern, Murtenstrasse 31, 3008 Bern, Switzerland; bastian.dislich@unibe.ch

**Keywords:** skin adnex, trichoblastic carcinoma, basal cell carcinoma, divergent trichoblastic differentiation, epidermal-mesenchymal transformation, COVID-19, SARS-CoV-2, spike

## Abstract

Basal cell carcinosarcomas (BCCSs) are rare, aggressive biphasic epithelial–mesenchymal neoplasms typically occurring in elderly patients. Their nonspecific clinical presentation may mimic inflammatory disorders, necessitating histopathological examination. We characterize a novel cutaneous BCCS with prominent trichoblastic differentiation, clinically mimicking ulcerative pyoderma gangrenosum. Although morphologically resembling trichoblastic carcinosarcoma (TBCS), the tumor retained a BCC-associated genetic signature, supporting a basal cell rather than a primary trichoblastic lineage. We therefore propose the provisional designation “basal cell carcinosarcoma with divergent trichoblastic differentiation (BCCS-DTB)”, suggesting acquired follicular lineage plasticity within a Hedgehog-driven, UV-mutagenized BCC clone. Shared molecular alterations across epithelial and sarcomatous components support monoclonality and an inside-out epithelial–mesenchymal transition (EMT) model, with progressive acquisition of trichoblastic and sarcomatous features. The patient’s young age, unusual tumor location, and development during the COVID-19 pandemic raise the hypothesis of additional environmental influences. Detection of associated spike protein and Snail expression is hypothesis-generating given their reported association with EMT. Further cases are required to validate the molecular profile for diagnostic and therapeutic relevance involving Hedgehog, FGFR2, and mTOR signaling.

## Simple Summary

Basal cell carcinosarcoma (BCCS) is a rare aggressive biphasic skin tumor that may clinically mimic inflammatory disorders. We report a BCCS with prominent trichoblastic differentiation in a young patient, presenting as an ulcer clinically diagnosed as pyoderma gangrenosum. Although the tumor showed striking trichoblastic morphology, molecular analysis revealed a characteristic BCC-associated profile, supporting a basal cell rather than primary trichoblastic origin. We propose the provisional designation “basal cell carcinosarcoma with divergent trichoblastic differentiation (BCCS-DTB)”. Shared genetic alterations in the epithelial and sarcomatous components support a monoclonal origin and an inside-out epithelial–mesenchymal transition (EMT) model, suggesting progressive acquisition of trichoblastic and sarcomatous features. The patient’s unusual age, tumor location, and development during the COVID-19 pandemic raise the possibility of additional environmental influences. Detection of associated spike protein and Snail expression is hypothesis-generating given their reported association with EMT. Further cases are required to validate this concept and its potential therapeutic implications.

## 1. Introduction

Carcinosarcomas are rarebiphasic epithelial and mesenchymal malignant neoplasms first named in visceral organs [1]. Primary cutaneous carcinosarcomas (cCSs) were first reported more than 90 years later and remain exceedingly uncommon, with fewer than 150 cases described, including fewer than 50 basal cell carcinosarcomas (BCCSs) [2,3].

In cCSs, the epithelial component may represent an epidermal or adnexal carcinoma. Among adnexal carcinomas, glandular differentiation is less common than follicular differentiation, the latter including tumors with basal cell or trichoblastic features [4,5]. The accompanying sarcomatous component typically consists of atypical spindle cells, with or without heterologous differentiation. Several pathogenetic models have been proposed to explain this biphasic phenotype, ranging from a reactive pseudosarcomatous response to the epithelial component (composition), through polyclonal collision of unrelated tumors (collision), multiclonal convergence of independently differentiated epithelial and mesenchymal components (convergence), and early monoclonal divergence from a common pluripotent progenitor (divergence/combination), to late monoclonal secondary epithelial-to-mesenchymal transformation (EMT) of the carcinoma (conversion) [6,7,8].

Clinically, BCCSs typically arise on highly chronically sun-damaged (high-CSD) skin of the head and neck in elderly men and present as rapidly growing, exophytic nodules or polypoid masses, often accompanied by ulceration, hemorrhage, or crusting [9,10]. Compared with conventional basal cell carcinomas (BCCs), BCCSs generally exhibit more rapid growth and may reach a larger size, although they are often clinically indistinguishable from conventional BCCs. The rarity of cCSs and their diverse clinical presentations make them challenging to recognize without histopathological examination. Here, we report a previously undescribed type of basal cell carcinosarcoma with trichoblastic comorphology that clinically mimicked an uncommon ulcerative pyoderma.

## 2. Skin Carcinosarcoma with Basaloid and Trichoblastic Differentiation: Clinics, Histomorphology, and Genetic Signature

**Figure 1:**
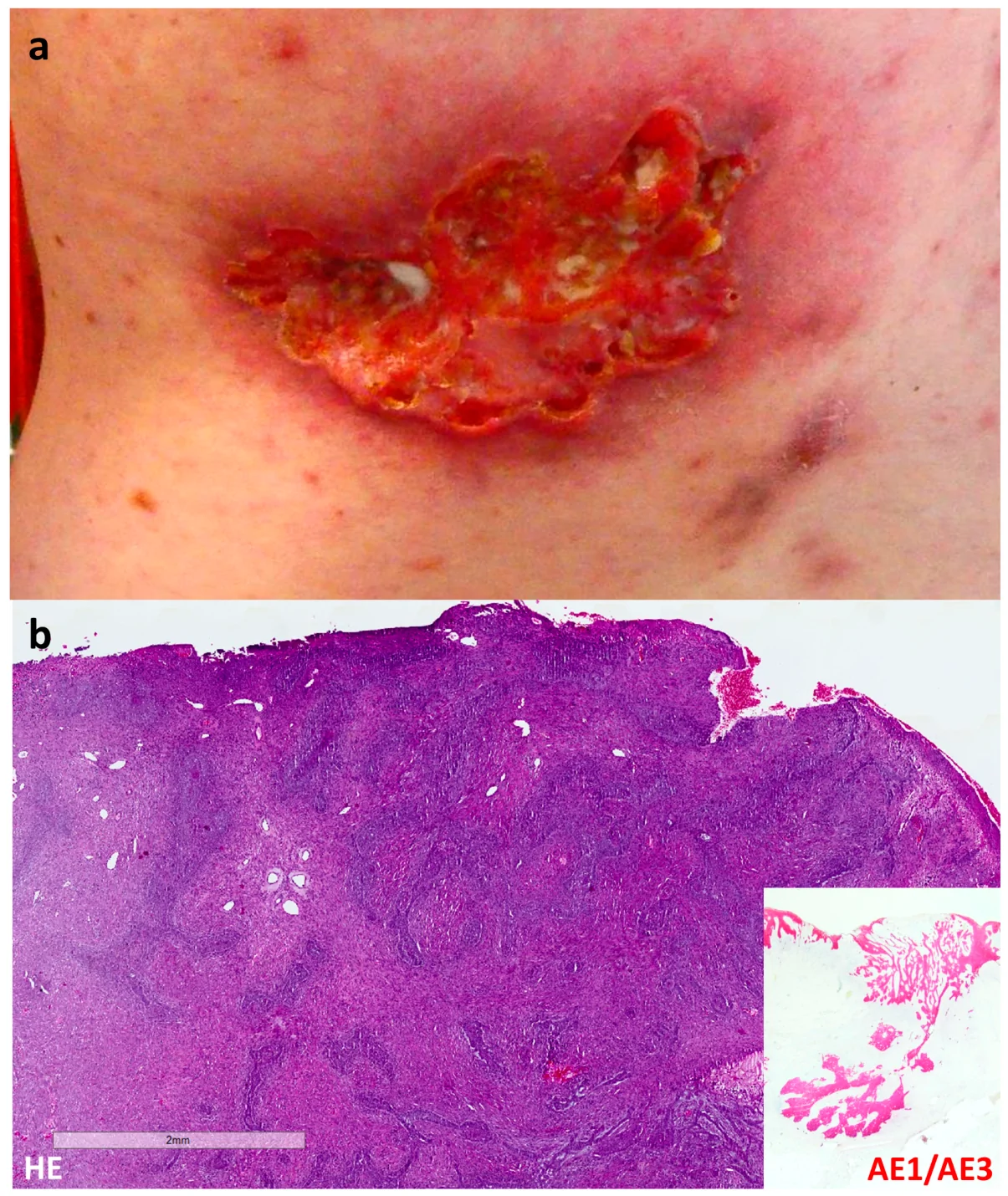
**Pyoderma gangrenosum-mimicking carcinosarcoma.** (**a**) clinically undermined ulcer with livid, sharply demarcated borders and central fibrinous deposits (9 cm × 4 cm × 0.5 cm); (**b**) histopathologically infiltrative biphasic epithelial–sarcomatous neoplasm composed of irregular, fenestrated, epidermis-connected branches of pleomorphic epithelial cells surrounded by pleomorphic stromal spindle cells (hematoxylin and eosin, H&E); inset: epidermis-connected epithelial silhouette of the carcinosarcoma highlighted by pancytokeratin AE1/AE3 immunohistochemistry. Original magnification, ×10.

A 42-year-old male presented with a painless, undermined, sharply demarcated ulceration on the left flank in an area of low chronic sun-damaged (low-CSD) skin, which had progressively enlarged for several months (Figure 1a). An initial superficial biopsy of the wound edge of the lesion revealed dermal suppurate infiltrates. The clinical diagnosis of pyoderma gangrenosum (PG) was initially supported by a positive Paracelsus Score (15/20) [11]. But the Delphi Consensus criteria were not fully met (1/1 major and 3/8 minor criteria), missing one additional minor criterion required for diagnosis [12]. This included only a partial response to immunosuppressive therapy consisting of predisolone (0.5 mg/kg body weight/d), topical halometasone/triclosan cream once daily for two weeks, followed by steroid-sparing treatment with systemic cyclosporine (2.5 mg/kg body weight/d) and tacrolimus 0.1% ointment.

**Figure 2:**
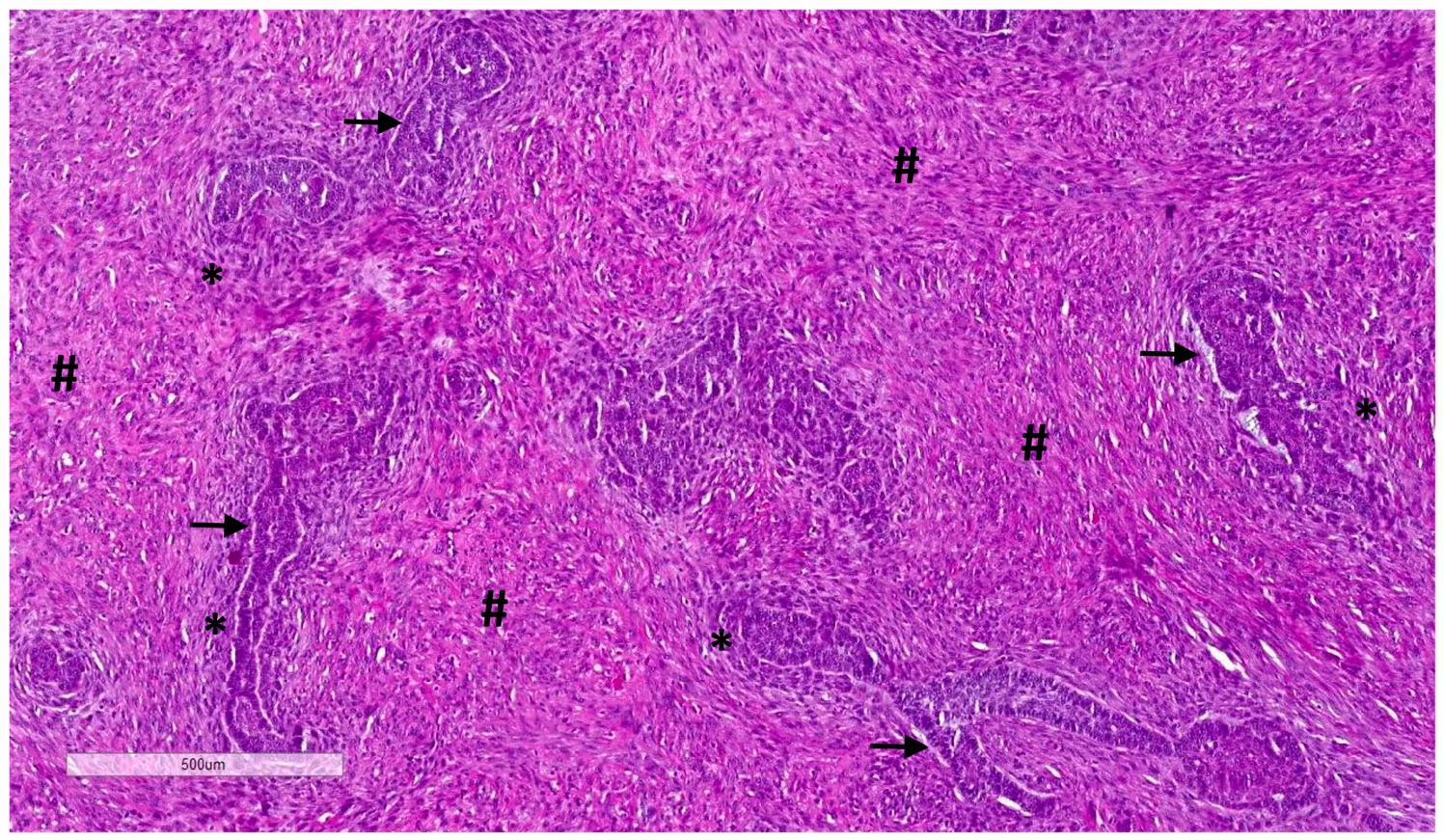
**Dual histomorphology of skin carcinosarcoma with basaloid and trichoblastic differentiation.** Epithelial compartment with atypical basaloid cells, peripheral palisading and periepithelial retraction artifacts (arrows), features characteristic of basal cell carcinomas; adjacent atypical cellular myxoid stroma forms rows of hyperchromatic cells tightly surrounding the epithelial aggregates and follicular papilla-like structures, resembling specialized trichogenic stroma (asterisks); more central mesenchymal compartment with diffuse monomorphic proliferation of atypical stromal cells (hash marks; hematoxylin and eosin, H&E).

After one year of clinical stability, the patient re-presented with a slowly progressive ulcerative lesion. Histopathological examination of a subsequent, deeper skin biopsy from the increasingly progressive lesion revealed an erosive, biphasic malignant epithelial–mesenchymal neoplasm connected to the epidermis (Figure 1b). The epithelial compartment consisted of fenestrated branches and strands of atypical, highly mitotic basaloid cells, with focal peripheral palisading, but without conspicuous panfollicular differentiation (Figure 2). The adjacent cellular, myxoid stromal compartment showed frequent mitoses, marked pleomorphism with nuclear atypia, and further periepithelial mucinous retraction artifacts, characteristic of basal cell carcinomas. In addition, the stoma formed rows of hyperchromatic cells tightly surrounding the epithelial aggregates and follicular papilla-like structures, both resembling specialized trichogenic stroma of trichoblastomas, albeit with pronounced cytological atypia. Further characteristic for trichoblastic tumors, the epithelial and stromal cells remained sharply demarcated without obvious transition between them. More distant from the epithelium, the atypical stromal cells proliferated in a diffuse spindle-shaped fashion in a vascular stroma, without heterologous elements.

**Figure 3:**
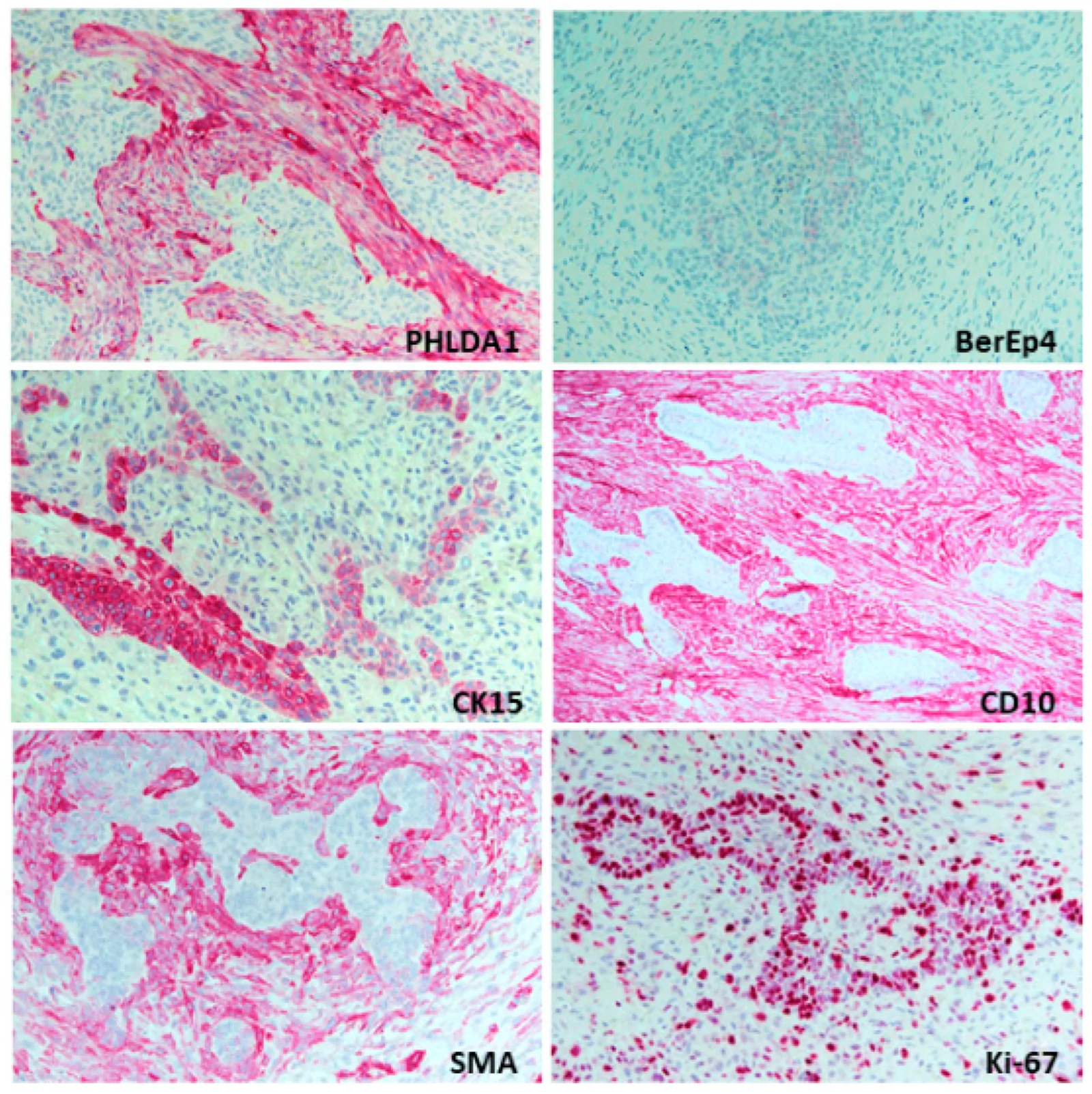
**Dual immunohistochemical profile of skin carcinosarcoma with basaloid and trichoblastic differentiation.** Immunohistochemical staining demonstrates markers of basal cell differentiation (faint central epithelial BerEP4 expression and epithelial Bcl2 positivity, see also Figure 4), together with markers supporting trichoblastic differentiation (PHLDA1 and CK15 positivity in the epithelial component and stromal CD10 positivity); the stromal component additionally shows myofibroblastic differentiation (SMA positive). Aberrant Ki-67 and p53 expression in both epithelial and sarcomatous compartments confirms high proliferative activity. Original magnification, ×200.

**Figure 4:**
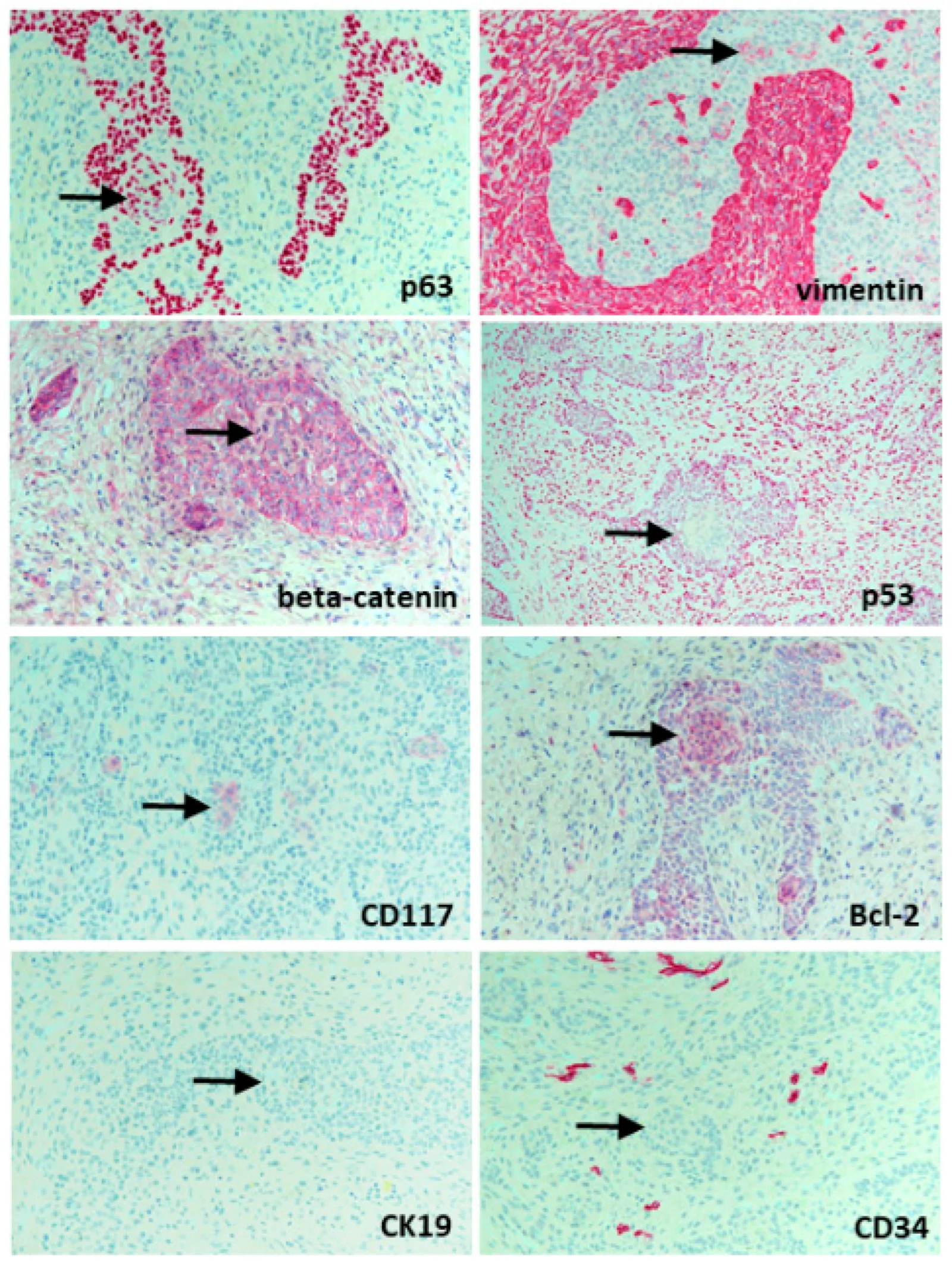
**Putative stem cell/progenitor cell markers highlight intraepithelial EMT-transitional cells in skin carcinosarcoma with basaloid and trichoblastic differentiation.** Arrows indicate intraepithelial islands of transitional cells, supporting an inside-out EMT conversion. These cells show an epithelial–mesenchymal transitional phenotype, with p63 and vimentin co-expression (see p63/vimentin double staining in Figure 5), accompanied by an incomplete putative stem cell marker profile (CD117 and Bcl2 positive; CD34 and CK19 negative), loss of membranous beta-catenin, and nuclear p53 expression. Original magnification, ×200.

Similarly, the immunohistochemical profile of the cCS revealed a mixed phenotype combining markers associated with basal cell carcinoma (BCC)-lineage (mainly Bcl-2 epithelial central positive and stromal negative, questionable BerEp4 faintly positive) with a marker showing a prominent trichoblastic lineage (PHLDA1 epithelial positive, CK15 epithelial positive, CD10 epithelial negative and stromal positive) (Figure 3 and Figure 4, antibody characteristics and staining patterns summarized in Supplementary Tables S1 and S2). The adjacent stromal compartment demonstrated spindle-cell heterologous myofibroblastic differentiation (SMA positive, desmin negative); both epithelial and mesenchymal compartments showed an abnormal proliferation rate (high Ki67 expression, aberrant p53 expression, mutational pattern).

**Figure 5:**
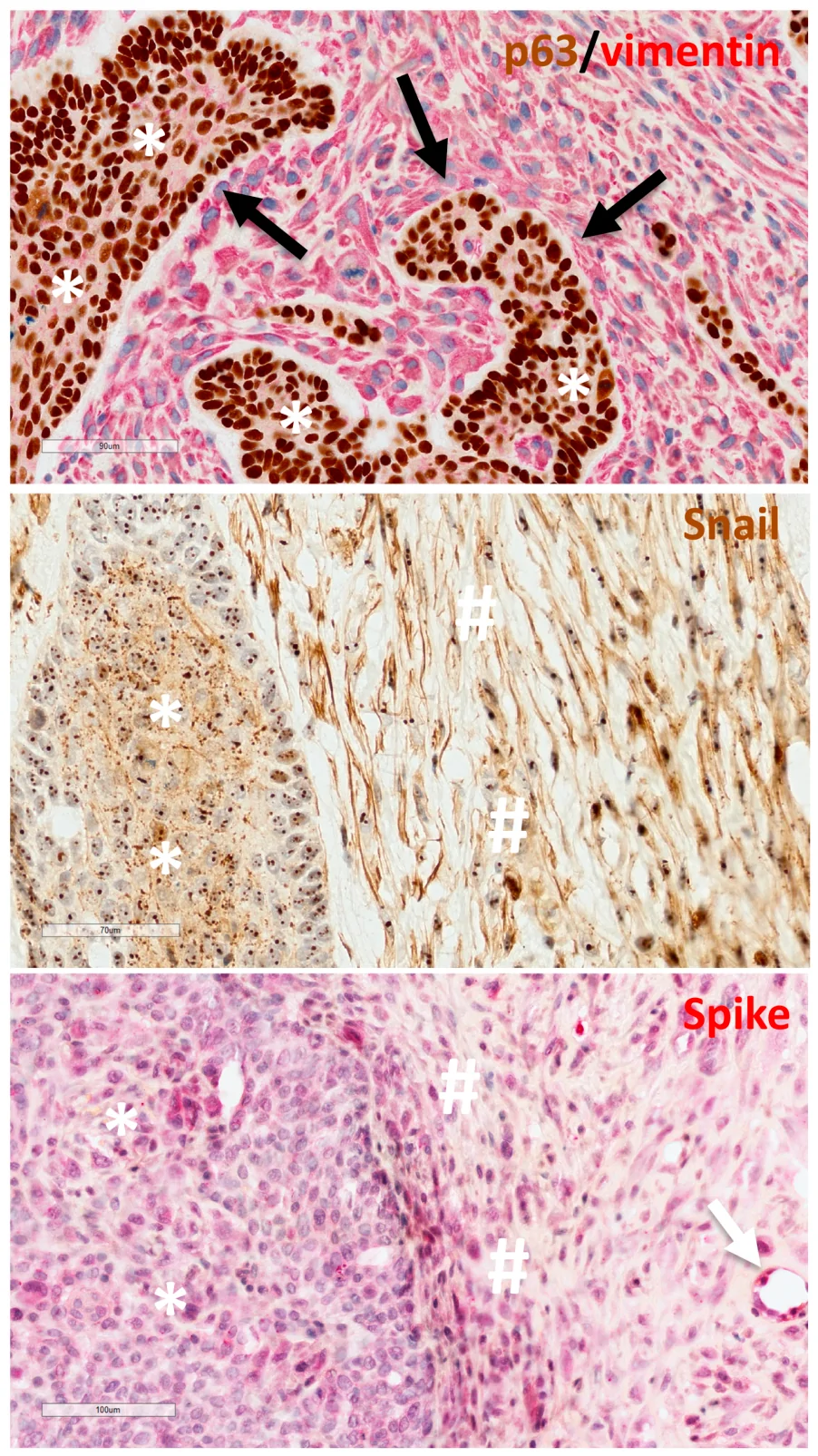
**EMT-related markers and spike protein mark intraepithelial transitional cells in skin carcinosarcoma with basaloid and trichoblastic differentiation.** p63/vimentin double staining identifies intraepithelial transitional cells (asterisks) co-expressing nuclear p63 and cytoplasmic vimentin, supporting an “inside-out” epithelial–mesenchymal transition (EMT). These cells show membrane, cytoplasmic, and nuclear Snail expression and slightly more prominent cytoplasmic spike staining, resembling the stromal sarcomatous component (hash symbols), whereas peripheral carcinoma cells show predominantly nuclear Snail and weaker spike staining. Tumor endothelial cells also express spike (white arrow); a corresponding basal cell carcinoma control stained for spike is shown in Supplemental Figure S1. The sharply demarcated epithelial–stromal interface (black arrows) reflects the characteristic “outside-in” architecture of trichoblastic tumors.

The clear-cut epithelial–mesenchymal divergence described for trichoblastic differentiation was evidenced by a sharply demarcated loss of epithelial markers, including CK15 and p63), with concomitant induction of mesenchymal markers, such as SMA and vimentin, and loss of membrane-bound beta-catenin in the sarcomatous compartment (Figure 3, Figure 4 and Figure 5). But a phenotypic transition between the two compartments was evident elsewhere, namely within multiple centrally located intraepithelial islands. These transitional areas revealed both a gradual loss of epithelial marker expression accompanied by progressive induction of mesenchymal markers (Figure 4 and Figure 5). Moreover, the EMT-associated markers beta-catenin and Snail demonstrated a progressive shift toward a mesenchymal staining pattern, paralleling the increasing loss of the epithelial phenotype as further indicated by a loss of nuclear p53. Additionally, these transitional cells expressed some stem cell-like markers (CD117 and Bcl2) but not others (CK19, CD34), further indicative of conversion.

Because of the tumor’s hybrid morphology, separate molecular analyses of the epithelial and sarcomatous components were performed on formalin-fixed, paraffin-embedded tissue. Tissue cores with needles of a diameter of 3 mm were obtained, and DNA and RNA were separately extracted from the epithelial and sarcomatous components, which had been manually delineated by a pathologist on an H&E reference section. Both tumor components were separately analyzed using the TruSight Oncology 500 gene panel (TSO500, Illumina, San Diego, CA, USA) and paired-end sequenced (NovaSeq 6000 platform, Illumina) at the Clinical Genomics Lab, Inselspital Bern. The panel interrogates exonic regions and splice sites of 523 genes as well as the most common pathogenic fusion transcripts in human cancers. A Variant Allele Fraction (VAF) detection threshold of 5% was applied, with a recommended minimum exon coverage of 50×- (deep sequencing).

Identical pathogenic mutations were identified in both epithelial and mesenchymal tumor components (tiers 1 and 2, Table 1), together with several shared and compartment-specific variants of undermined significance (tiers 3 and 4). The shared oncogenic alterations were classified into the following: (1) an activating gain-of-function (GOF) mutation with a strong UV-damage signature (TERT promoter); (2) a UV-linked GOF mutation in an oncogene (FGFR2); and (3) loss-of-function (LOF) mutations in tumor suppressor genes either UV-dependent (TP53), mixed (PTCH1) or not linked to UV-damage (TSC1, CREBBP) [7,13]. The molecular profile included canonical BCC-associated driver alterations involving PTCH1 and the TERT promoter. PTCH1 mutations have previously been reported in basal cell carcinosarcoma (BCCS), whereas the TERT promoter alteration has not, to our knowledge, been previously described in this setting. A non-hotspot TP53 mutation, previously reported in BCCs but not in BCCSs, was also detected. Additional rare or uncommon alterations involved CREBBP, TSC1, and FGFR2, with variable previous associations with BCCs and BCCSs. In contrast, genetic alterations proposed to support a trichoblastic lineage, including mutations involving CDKN2A, CTNNB1, RAS-pathway genes, or SUFU, were not detected.

Given the patient’s relatively young age and the presence of PTCH1 mutations, basal cell nevus syndrome was considered. However, no clinical features, including multiple basal cell carcinomas or palmar pits, or radiological findings, such as intracranial calcifications or odontogenic jaw cysts, were present to support this diagnosis. Notably, the tumor developed toward the end of the COVID-19 pandemic, during which the patient had been exposed to the SARS-CoV-2 virus and preventive measures, including two doses of the BNT162b2 (Comirnaty^®^, BioNTech SE, Mainz, Germany) vaccine. Compared with BCC control, immunohistochemical analysis of the carcinosarcoma demonstrated spike immunoreactivity in vascular endothelial cells as well as in both the epithelial and mesenchymal tumor components, suggesting prior exposure to spike protein (Figure 5 and Figure S1). The family medical history was unremarkable.

MRI demonstrated a tumor mass infiltrating the subcutis, while subsequent PET-CT staging revealed no evidence of metastatic disease. Following the recommendation of the multidisciplinary tumor board, the carcinosarcoma was completely excised with 2 cm surgical margins, and the defect was reconstructed with a split-thickness skin graft. Postoperative imaging after one year showed no radiological evidence of recurrence. The subsequent clinical course remained unremarkable, with no evidence of recurrence during six-monthly follow-up over a total period of four years.

## 3. Discussion

Primary cutaneous carcinosarcomas (cCSs) are rare biphasic neoplasms composed of malignant epithelial and mesenchymal components. Clinically, they may mimic inflammatory dermatoses. To our knowledge, this is the first cCS reported as a clinical mimic of pyoderma gangrenosum (PG), presenting with undermined inflammatory ulcer margins and a positive Paracelsus Score, but without fulfillment of the Delphi Consensus minor criteria (Figure 1a). The initial diagnostic uncertainty and partial response to immunosuppression may have contributed to delayed re-biopsy and subsequent clinical progression (Figure 1b).

But the unusual biology of this skin carcinosarcoma (cCS) was more remarkable than the rarity.

First, morphology and immunohistochemistry demonstrated a hybrid phenotype combining conventional basaloid/BCC features with prominent trichoblastic differentiation (Figure 2, Figure 3, Figure 4 and Figure 5; Supplementary Table S2). The basaloid component showed epidermal connection, peripheral palisading, and periepithelial retraction, whereas epithelial fenestration, papilla-like cellular stroma, and sharply demarcated epithelial–mesenchymal interfaces indicated trichoblastic differentiation. Thus, morphologically, the tumor occupies an intermediate position between conventional basal cell carcinosarcoma (BCCS) and trichoblastic carcinosarcoma (TBCS).

Second, genetic analysis was decisive for histogenetic classification. The tumor harbored a canonical BCC lineage signature, including PTCH1 and TERT alterations (Table 1), in the absence of characteristic alterations associated with trichoblastic neoplasia, including CDKN2A, CTNNB1, RAS-pathway, or SUFU alterations.

These findings argue against a molecularly programmed trichoblastic phenotype analogous to that of a true TBCS [14,15]. Together with the mixed UV mutational profile, these findings favor a classification within the BCCS spectrum. We therefore propose the designation basal cell carcinosarcoma with divergent trichoblastic differentiation (BCCS-DTB) for this morphologically distinct phenotype; see further discussion below.

**Figure 6:**
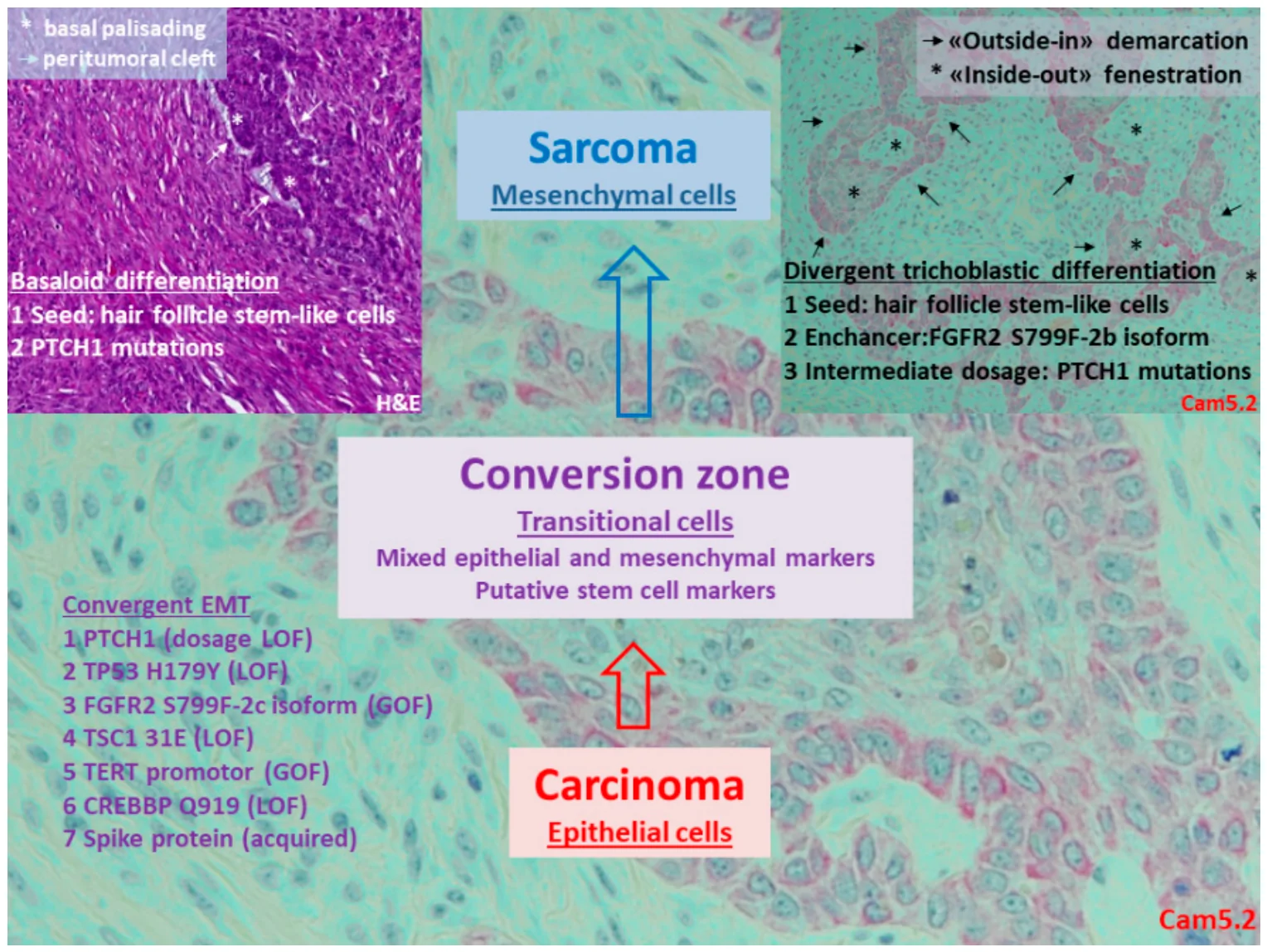
**From carcinoma to sarcoma: proposed model of divergent trichoblastic differentiation and inside-out sarcomatous conversion.** Compartment-shared genetic alterations drive both basal cell and trichoblastic differentiation and promote EMT-mediated conversion. EMT-transitional cells emerge centrally within the epithelial compartment (inside-out conversion) and progressively acquire a mesenchymal phenotype, while the peripheral epithelial–stromal borders remain sharply demarcated (outside-in architecture). Skin carcinosarcoma may therefore represent a transitional neoplasm in the evolution from carcinoma to sarcoma (low-weight cytokeratins, Cam5.2); inset: hematoxylin and eosin, H&E. Original magnification, ×200.

The pronounced trichoblastic differentiation despite the absence of a canonical trichoblastic mutational profile may reflect the convergence of developmental and signaling mechanisms (Figure 3 and Figure 6, Table 1):

1. Trichoblastic seed: Hair-follicle stem or stem-like cells of origin may intrinsically provide a trichoblastic developmental program, without requiring additional lineage-specifying mutations [14,16,17].
2. Trichoblastic dosage: The detected PTCH1 splice-site mutation may result in a hypomorphic PTCH1 allele, producing intermediate rather than maximal Hedgehog pathway activation and thereby favoring a trichoblastic phenotype [17,18].
3. Trichoblastic enhancer: The cCS-associated novel gain-of-function FGFR2 S799C mutation may promote aberrant follicular differentiation through RAS and PI3K signaling, potentially through constitutive activation of the FGFR2b isoform (Table 1) [19]. FGFR2 signaling interacts with Hedgehog and bone morphogenetic protein pathways, all of which are critically involved in hair-follicle development and differentiation [20,21].
4. Additional epigenetic or transcriptional alterations: Unmeasured epigenetic or transcriptomic changes may contribute to the phenotype but remain beyond the resolution of targeted DNA sequencing. Interestingly, sporadic trichoblastic neoplasms have been reported to exhibit a high tumor mutational burden, as observed in our case, but typically lack a UV mutational signature, in contrast to the present tumor [7].

Comparison of the distribution of the detected genetic alterations revealed identical molecular profiles in the epithelial and sarcomatous compartments (Table 1), supporting a shared monoclonal origin. Although this finding is compatible with either divergent differentiation or epithelial–mesenchymal conversion, the absence of sarcoma-specific alterations that could account for the heterologous mesenchymal phenotype, the myofibroblastic or even the pronounced trichoblastic differentiation, argues against a simple divergence model [22,23]. Moreover, the divergence hypothesis requires several additional assumptions, including persistence of a primitive stem-cell population into adulthood followed by selective genetic alteration and subsequent divergent differentiation into epithelial and mesenchymal lineages [8]. Conversely, the BCC lineage signature and the progressive reduction in the sarcomatous component toward the tumor periphery favor an epithelial origin followed by mesenchymal conversion.

We therefore hypothesize an EMT-based inside-out model, in which epithelial cells undergo progressive transition toward a mesenchymal phenotype while retaining the original BCC-associated genetic background (Figure 4, Figure 5 and Figure 6). The observed p63/vimentin-positive transitional cells and Snail expression pattern provide morphological support for this concept. Conversely, a mesenchymal-to-epithelial transition (MET) could theoretically be considered; however, although epithelial dedifferentiation has rarely been described in primary sarcomas, convincing examples of such a process resulting in organized epithelial architecture have not been established in cutaneous sarcomas [24]. Instead, several features of the present molecular profile provide additional, albeit hypothesis-generating, support for an EMT-based mechanism. All six detected alterations could potentially contribute to pathways involved in epithelial–mesenchymal conversion. PTCH1 loss may represent an initiating event, while TP53 H179Y could facilitate subsequent genomic instability and progression. FGFR2 S799C may enhance Snail1 expression through altered FGFR2b/FGFR2c signaling, whereas TSC1 E31 loss has been associated with increased Snail1 expression and reduced E-cadherin. The TERT promoter mutation may facilitate nuclear beta-catenin signaling, while CREBBP Q919 loss could induce epigenetic alterations favoring mesenchymal gene expression. Collectively, these alterations provide a plausible molecular framework for progressive EMT and sarcomatous transformation (Figure 4, Figure 5 and Figure 6) [25,26,27,28].

The patient’s young age, the tumor occurrence in low chronic sun-damaged skin, and the absence of features suggestive of a nevoid BCC syndrome raise the possibility of additional acquired influences. The temporal association with the COVID-19 pandemic is of interest, particularly given reports of more advanced tumor stages during this period [29]. Experimentally, the spike protein drives EMT, first characterized in SARS-CoV-2-associated pulmonary fibrosis, and subsequently through mechanisms involving Snail activation [30]. Spike-mediated EMT has also been implicated in lung cancer models, where it may promote tumor progression and therapeutic resistance [31]. Whether such mechanisms or associated immunmodulation could contribute to progression of a conventional BCC toward a carcinosarcomatous phenotype remains speculative. The detection of spike protein within the tumor associated with Snail provides an additional observation of potential interest in this context (Figure 5) but does not establish causality. Further, there is currently no established evidence linking SARS-CoV-2 spike protein to trichoblastic differentiation or follicular lineage signaling.

Further, this case may also provide a conceptual framework for understanding EMT in trichoblastic tumors. Trichoblastic differentiation is characterized by sharply demarcated epithelial and stromal compartments without obvious transition (outside-in) (Figure 2, Figure 3, Figure 4, Figure 5 and Figure 6) [6,7,32,33]. In contrast to reports favoring an outside-in transition, we propose an inside-out model, in which EMT is initiated centrally within the epithelial compartment. Here, transitional cells acquire a stem cell-like phenotype and progressively lose epithelial while gaining mesenchymal marker expression. Subsequent expansion may result in epithelial fenestration and formation of multiple tumor islands with trichoblastic-like stromal organization (Figure 6). Such a model could reconcile the apparent discrepancy between an EMT-based conversion mechanism and the sharply separated epithelial and stromal compartments characteristic of trichoblastic differentiation. The shared molecular profile of the epithelial and sarcomatous components and the presence of multiple potentially EMT-related alterations further support this concept. We therefore hypothesize that progressive conversion of the epithelial compartment may ultimately result in complete sarcomatous transformation, with BCCS representing a transitional stage in this process. This hypothesis is compatible with a reported PTCH1-mutated BCCS that subsequently persisted as a pure sarcoma without a detectable epithelial component [15].

This proposed model also distinguishes BCCS-DTB from sarcomatoid BCC. Sarcomatoid BCC is regarded as an incipient/incomplete EMT, with spindle cells adjacent to conventional BCC that often retain keratin expression and lack heterologous differentiation. In contrast, the sarcomatous component of carcinosarcoma shows complete loss of epithelial differentiation, with vimentin-positive, keratin-negative cells and, in some cases, heterologous elements [9]. Although the WHO recognizes sarcomatoid BCC as a manifestation of incipient BCC-derived EMT, no specific terminology currently exists for a BCC-derived carcinosarcoma representing more advanced or complete EMT [34]. The converse situation applies to malignant trichoblastic tumors. WHO-defined trichoblastic carcinosarcoma (TBCS) is a biphasic malignant tumor with advanced follicular epithelial and trichoblastic components. Although focal peripheral palisading and stromal retraction may occur in TBCS, these features are not entirely specific for the BCC lineage [35]. But molecularly, TBCS generally lacks the UV mutational signature and PTCH1 alterations characteristic of BCC-lineage tumors and instead may harbor shared alterations such as CDKN2A and CTNNB1 across both compartments, supporting origin from a trichoblastic progenitor. However, a corresponding lesion with incipient EMT is not defined for trichblastic tumors.

In the present case, the combination of (i) a UV signature, (ii) a PTCH1 splice-site-mutant clone characteristic of BCC, and (iii) prominent trichoblastic morphology with organized epithelial–stromal interactions supports classification as basal cell carcinosarcoma with divergent trichoblastic differentiation (BCCS-DTB). This designation distinguishes the tumor from conventional biphasic BCCSs, monophasic sarcomatoid BCCs, and WHO-defined TBCSs, while recognizing its hybrid phenotype.

The term “divergent trichoblastic differentiation” is intended to indicate an additional phenotypic shift within a clonally BCC-derived carcinosarcoma rather than primary trichoblastic lineage. This distinction is relevant because “BCC with follicular/trichoblastic differentiation” already describes conventional BCCs showing focal histological recapitulation of follicular structures. In contrast, BCCS-DTB denotes lineage plasticity within a biphasic malignant clone accompanied by complete mesenchymal differentiation rather than merely focal follicular morphology in an otherwise conventional BCCS. Given that the proposed classification is based on a single case, validation in additional tumors is essential.

## 4. Conclusions

**Figure 7:**
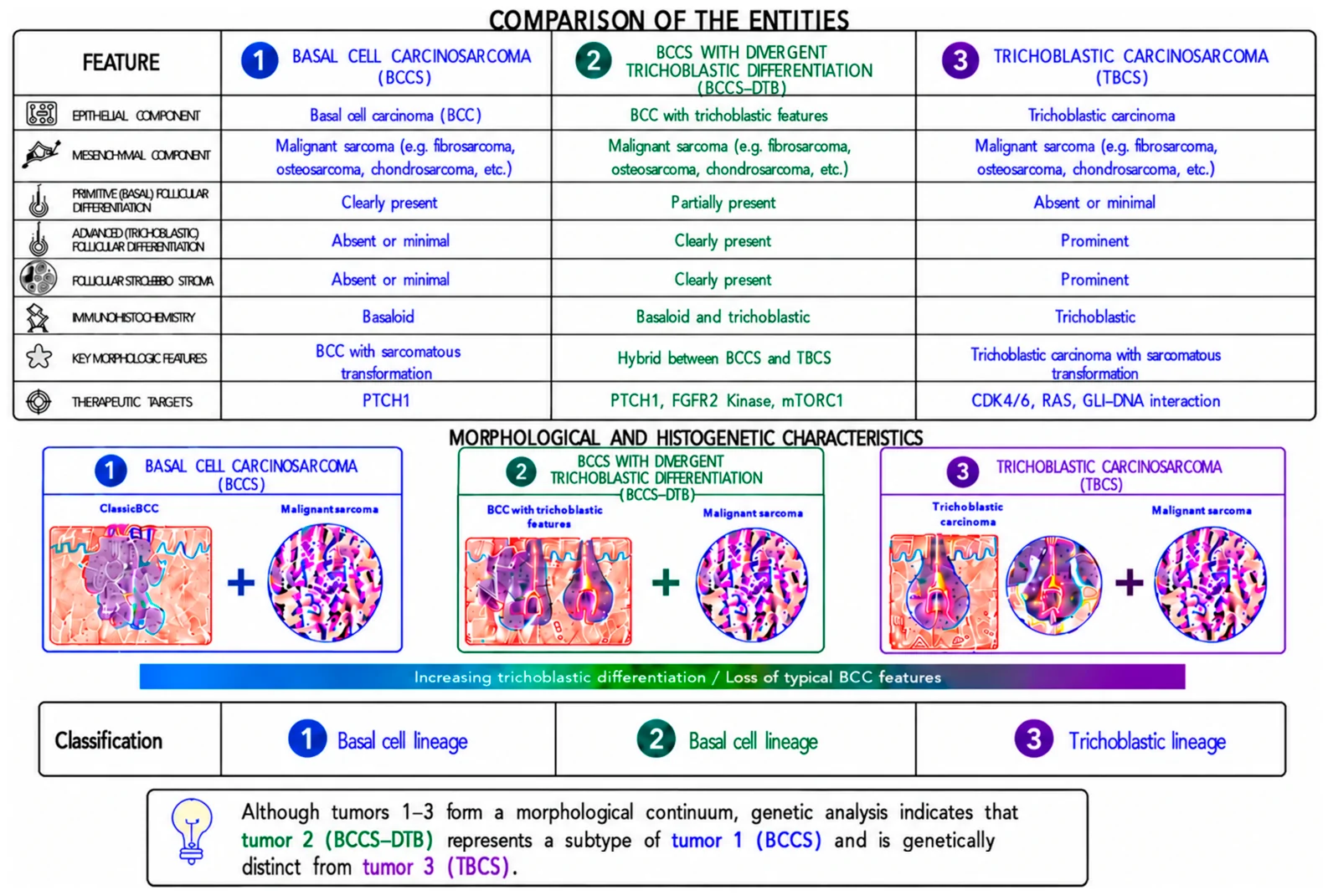
**Proposed classification of basal cell carcinosarcoma with divergent trichoblastic differentiation (BCCS-DTB) in comparison with conventional basal cell carcinosarcoma (BCCS) and the unrelated trichoblastic carcinosarcoma (TBCS).** This graph summarizes the lineage, morphology, immunophenotype, and molecular targets of these biphasic cutaneous neoplasms. Although proposed BCCS-DTB closely resembles TBCS morphologically, their genetic profiles are distinct: BCCS-DTB retains the molecular signature of BCC and is therefore genetically related to conventional BCCS, whereas TBCS represents a genetically distinct follicular-lineage neoplasm. This model supports BCCS-DTB as a morphologically TBCS-like but genetically BCC-derived transitional entity within the basal cell lineage, while maintaining TBCS as a separate follicular-lineage neoplasm, each offering different therapeutic targets.

This case highlights the diagnostic challenge posed by primary cutaneous carcinosarcoma, which may clinically mimic inflammatory ulcerative disorders such as pyoderma gangrenosum. The combined morphologic and molecular findings support basal cell carcinosarcoma with divergent trichoblastic differentiation (BCCS-DTB), characterized by a BCC-associated genetic profile and prominent trichoblastic morphology (Figure 7). Shared molecular alterations across the epithelial and sarcomatous components, together with epithelial–mesenchymal transitional phenotypes, support a monoclonal origin and favor progressive epithelial-to-mesenchymal conversion over divergent tumor development. We therefore propose an inside-out EMT model, in which a BCC-derived epithelial clone progressively acquires trichoblastic and ultimately sarcomatous features. The patient’s young age, unusual tumor location, and temporal association with the COVID-19 pandemic further raise the possibility that additional environmental or inflammatory factors may have contributed to tumor development, although such a relationship remains speculative.

The molecular profile may also identify potential therapeutic vulnerabilities. Beyond targeting the BCC-associated PTCH1/Hedgehog pathway with SMO inhibitors in advanced disease, the FGFR2 S799C alteration provides a rationale for exploring FGFR-directed approaches, while alterations affecting mTOR signaling may represent an additional therapeutic target. However, these potential treatment strategies remain speculative and require functional validation, particularly given the complex EMT phenotype and the absence of clinical treatment data.

As these conclusions are based on a single case, BCCS-DTB should currently be regarded as a descriptive, hypothesis-generating concept rather than an established entity. Identification and comprehensive molecular characterization of additional cases will be required to determine its reproducibility, histogenetic basis, therapeutic vulnerabilities, and clinical significance.

## Figures and Tables

**Table 1:** Summary of pathogenic genetic alterations identified in both the epithelial and the mesenchymal component of the skin carcinosarcoma with basaloid and trichoblastic differentiation. An asterisk (*) denotes a stop codon.

Alteration	Gene	Amino Acid	cDNA	VAF Epithelial (%)	VAF Mesenchymal (%)
Mutation	CREBBP	p.Q919*	c.2755C>T	0.3426	0.3496
Mutation	FGFR2	p.S799Ffs*22	c.2395dup	0.3448	0.3443
Splice Site Mutation	PTCH1	NA	c.1603-1_1603delinsAA	0.5727	0.5106
Promoter Mutation	TERT Promoter	NA	g.1295250C>T	0.5	0.4828
Mutation	TP53	p.H179Y	c.535C>T	0.5558	0.4948
Mutation	TSC1	p.E31*	c.91G>T	0.4652	0.5162

## Data Availability

Data are contained in the manuscript.

## Supplementary Materials

The following supporting information can be downloaded at: https://www.mdpi.com/article/10.3390/dermatopathology13030045/s1 Table S1: Antibody panel and technical parameters for immunohistochemical analysis; Table S2: Immunophenotypic profile of the epithelial, transitional, and sarcomatous components of the tumor; Figure S1: Spike protein expression in basal cell carcinoma control. Basal cell carcinoma cells (asterisks), stromal cells (hash symbols), and surrounding vasculature show no specific spike protein staining. Insets show hematoxylin and eosin staining (H&E) and a secondary-antibody control (ab-control).
