# Supplementary material for: Skin Basal Cell Carcinosarcoma in a Trichoblastic Guise—Molecular Characterization of a Novel Entity Mimicking a Pyoderma Gangrenosum
Source: Dermatopathology (Basel). 2026 Sep 16;13(3):45. doi: 10.3390/dermatopathology13030045 (PMC13605686; doi:10.3390/dermatopathology13030045)

Supplemental material

Suppl. Table S1. **Antibody panel and technical parameters for immunohistochemical analysis**

Antibody	target	manufacturer	reference number	clone	RRID	dilution	incubation time	antigen retrieval	detection system
Anti-human AE1/AE3	low and high molecular weigh cytokeratins (56, 65 to 67 kD)	Dako	M351501-2	AE1/AE3	RRID:AB_2631307	*1:500	15 min	ER2 (30 min) 95°C	Bond Polymer Refine Red Detection (Leica-DS9390)
Anti-human Bcl-2	B-cell lymphoma 2, Bcl-2	Leica	PA0117	100/D5	RRID:AB_10555268	Bond RTU	15 min	ER2 (20 min) 100°C	Bond Polymer Refine Red Detection (Leica-DS9390)
Anti-human BerEp4	Ep-CAM/Epithelial Specific Antigen, BerEp4	DBS (Diagnostic Biosystems)	PDM131	BerEp4	no result	RTU	60 min	ER1 (30 min) 100°C	Bond Polymer Refine Red Detection (Leica-DS9390)
Anti-human beta-catenin	beta-catenin	Cell Marque	224M	14	RRID:AB_1158971	*1:500	15 min	ER2 (30 min) 100°C	Bond Polymer Refine Red Detection (Leica-DS9390)
Anti-human CD10	cluster of differentiation 10, CD10	Leica	PA0270	56C6	incorrectly deposited	RTU	15 min	ER2 (20 min) 100°C	Bond Polymer Refine Red Detection (Leica-DS9390)
Anti-human CD117	cluster of differentiation CD117	Novocastra Leica	NCL-L-CD117-032	T595	RRID:AB_563487	*1:200	60 min	ER2 (20 min) 95°C	Bond Polymer Refine Red Detection (Leica-DS9390)
Anti-human CD34	cluster of differentiation 34, CD34	Leica	PA0212	QBEnd/10	incorrectly deposited	Bond RTU *1:10	15 min	ER2 (20 min) 100 °C	Bond Polymer Refine Red Detection (Leica-DS9390)
Anti-human CK15	intermediate filament keratin 15	DBS (Diagnostic Biosystems)	Mob 305	LKH15	RRID:AB_2132884	*1:25	15 min	ER1 (10 min) 100°C	Bond Polymer Refine Red Detection (Leica-DS9390)
Anti-human CK19	intermediate filament keratin 19	Leica	PA0799	B170	RRID:AB_10554292	Bond RTU *1:5	15 min	ER2 (20 min) 100 °C	Bond Polymer Refine Red Detection (Leica-DS9390)

Anti-human Ki67	Kiel 67, Ki-67	Leica	PA0230	K2	RRID:AB_2341199	Bond RTU	15 min	ER2 (20 min) 100°C	Bond Polymer Refine Red Detection (Leica-DS9390)
Anti-human p53	protein 53, p53	Dako	IR61661-2	DO-7	RRID:AB_3669092	RTU *1:4	15 min	ER2 (20 min) 95°C	Bond Polymer Refine Red Detection (Leica-DS9390)
Anti-human p63	protein 63, p63	Bio SB	BSB3607	4A4	RRID:AB_3722733	*1:100	15 min	ER2 (30 min) 100°C	Bond Polymer Refine Red Detection (Leica-DS9390)
Anti-human PHLDA1 (TDAG)	Pleckstrin Homology-Like Domain Family A Member 1, PHLDA1	Santa Cruz	sc-23866	RN-6E2	RRID:AB_628117	*1:200	60 min	ER2 (10 min) 100°C	Bond Polymer Refine Red Detection (Leica-DS9390)
Anti-human SMA	smooth muscle actin,SMA	Leica	PA0943	αsm1	RRID:AB_10555985	Bond RTU	15 min	ER1 (10 min) 100°C	Bond Polymer Refine Red Detection (Leica-DS9390)
Anti-human Vimentin	intermediate filament vimentin	Novocastra Leica	NCL-L-VIM-V9	V9	RRID:AB_564055	Novocastra RTU *1:100	15 min	ER2 (10 min) 100°C	Bond Polymer Refine Red Detection (Leica-DS9390)
Anti-human Snail	Snail	Novus	NBP1-19529	poly rb	no result	*1:200	30 min	ER2 (30min) 95°C	Bond Polymer Refine Detection (Leica-DS9800)

Suppl. Table S2. **Immunophenotypic profile of the epithelial, transitional, and sarcomatous components of the tumor.**

antibody	carcinoma	transitional area	sarcoma	usage
AE1/AE3	cytoplasmic	weak cytoplasmic	negative	epithelial marker
Cam 5.2	cytoplasmic	weak cytoplasmic	negative	epithelial marker
p63	nuclear	weak nuclear	negative	epithelial marker
Bcl2	cytoplasmic	strong cytoplasmic positive	negative	basal cell phenotype, putative stem cell/ progenitor cell marker
BerEp4	negative	faint cytoplasmic positive	negative	basal cell phenotype
PHLDA1	cytoplasmic	weak cytoplasmic positive	negative	trichoblastic phenotype
CK15	cytoplasmic	weak cytoplasmic positive	negative	trichoblastic phenotype
CD10	negative	negative	cytoplasmic	trichoblastic phenotype
vimentin	negative	weak cytoplasmic positive	cytoplasmic	mesencymal marker
SMA	negative	faint cytoplasmic positive	cytoplasmic	heterologous mesencymal phenotype
beta-catenin	membrane	membrane/cytoplasmic/nuclear	membrane/cytoplasmic/nuclear	EMT-accociated marker
Snail	nuclear	membrane/cytoplasmic/nuclear	membrane/cytoplasmic/nuclear	EMT-associated marker
CD117	negative	weak cytoplasmic positive	negative	putative stem cell/ progenitor cell marker
CK19	negative	negative	negative	putative stem cell/ progenitor cell marker
CD34	negative	negative	negative	putative stem cell/ progenitor cell marker
Ki67	nuclear	nuclear	nuclear	proliferation marker
p53	nuclear	negative	nuclear	proliferation marker-mutational pattern

Supplementary Figure S1. **Spike protein expression in basal cell carcinoma control.** Basal cell carcinoma cells (asterisks), stromal cells (hash symbols), and surrounding vasculature show no specific spike protein staining. Insets show hematoxylin & eosin staining (H&E) and a secondary-antibody control (ab-control).

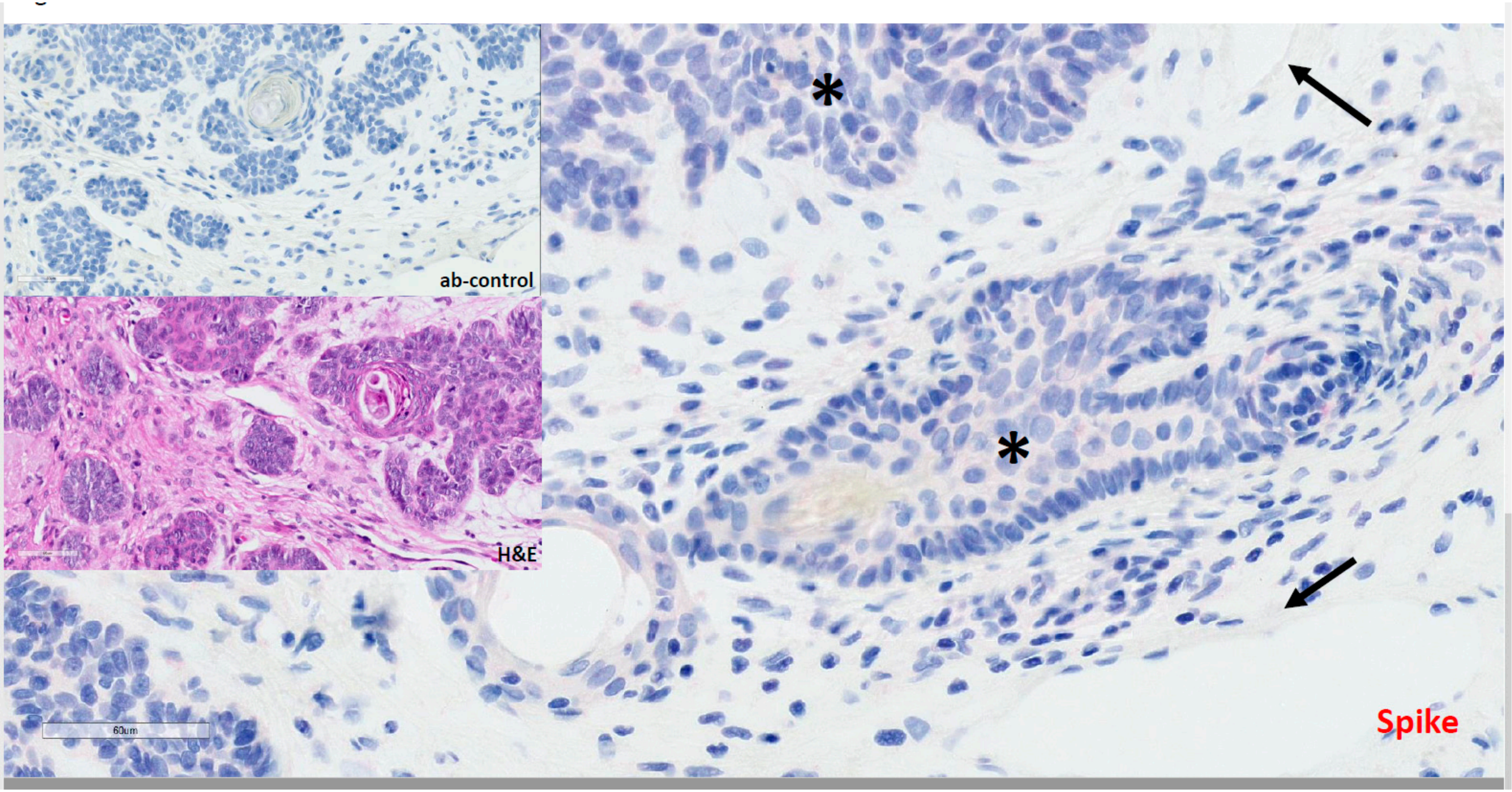

Supplement: Supplementary file 1 [file dermatopathology-13-00045-s001.zip › dermatopathology-4459554-supplementary.pdf]
